# Lumbar Sympathetic Block Leading to Increased Arterial Diameter and Blood Flow: A Mechanism of Therapeutic Benefit

**DOI:** 10.7759/cureus.61755

**Published:** 2024-06-05

**Authors:** Zachary Dickey, Navneet Sharma

**Affiliations:** 1 Physical Medicine and Rehabilitation, Edward Via College of Osteopathic Medicine, Monroe, USA; 2 Physical Medicine and Rehabilitation, Green Clinic, Ruston, USA; 3 Rehabilitation Medicine, Ruston Regional Specialty Hospital, Ruston, USA

**Keywords:** autonomic neurology, physical medicine and rehabilitation (pm&r), interventional spine, complex regional pain, visceral somatics, chronic pain management, peripheral arterial diseases, sympathetic ganglion, spinal neuromodulation, lumbar sympathetic block

## Abstract

Lumbar sympathetic blocks (LSBs) have long been used for the treatment of chronic lower extremity pain and for conditions such as complex regional pain syndrome (CRPS). With a better understanding of the autonomic nervous system and its function, these blocks have grown in their utility. Through this growth, however, our understanding of sympathetic-mediated pain is still vaguely understood. Here, we present a case of a patient who underwent a point-of-care ultrasound (POCUS) before and after an LSB, and we were able to show significant dilation of the posterior tibial artery (PTA) following the block. We propose that this arterial dilation plays a mechanistic role in providing pain relief to patients who undergo LSB. This increased blood flow can not only enhance healing properties to surrounding tissues but also allow for nitric oxide to play potential regulatory roles in pain pathways. Here, we also review potential mechanisms of the amelioration of sympathetic-mediated pain as well as the potential utilization of LSBs and neuromodulation in treating visceral pathologies through a better understanding of visceral somatic relationships.

## Introduction

Sympathetic nerve blocks are growing in their utility as we better understand pain and its relationship to the autonomic nervous system; however, the mechanism of their benefit is still poorly understood. Lumbar sympathetic blocks (LSBs) are currently being used for a wide range of conditions such as complex regional pain syndrome (CRPS), phantom limb pain, painful vascular insufficiencies, neuropathic pain, vascular pain, hyperhidrosis, shingles, Raynaud's syndrome, ulcers/wound care, and cancer-induced pain [1]. LSBs are proposed to decrease norepinephrine from sympathetic nerve endings, decrease sensory and vasomotor nerve fibers associated with pain, and increase blood flow [2]. While these mechanisms are still very poorly understood, we aimed to investigate the role of vasodilation in the lower extremity following sympathetic blocks to explore the potential therapeutic benefit via increased blood flow. Furthermore, we emphasize the importance of understanding autonomic structure and function and the relationship of various spinal levels to different viscera when performing an LSB.

The autonomic nervous system consists of both the parasympathetic and sympathetic nervous system (SNS). The SNS is what is commonly referred to as the "flight or fight" response. It is composed exclusively of efferent neurons that divide into a right and left sympathetic trunk extending from the skull to the coccyx paravertebrally [3,4]. These various ganglia consist of pre-ganglionic fibers that synapse with post-ganglionic fibers before their ascent or descent to various visceral organs [3]. Interestingly, the fibers of the SNS only span from the levels of T1-L2, while the parasympathetic nervous system stems from the vagus nerve and the pelvic splanchnic ganglion S2-S4 [5].

Sympathetic-mediated pain can stem from various causes that disrupt innate homeostasis. Pathologic mechanisms that overstimulate the SNS can put the body under increased oxidative stress and stimulate various pain pathways; however, the exact mechanism of sympathetic-mediated pain is poorly understood. The majority of sympathetic-mediated pain cases and the current understanding of this type of pain center around a condition known as CRPS. CRPS is a neuropathic condition that consists of hyperalgesia, allodynia, and vasomotor, sudomotor, motor, and trophic changes [6]. The basis of the CRPS association with sympathetic-induced pain is through its observed skin color changes, hyperhidrosis, and changes in extremity temperature [7]. CRPS can be treated with various modalities, one of which includes a sympathetic nerve block.

The sympathetic nerve block consists of blocking the stellate ganglion for upper extremity-related symptoms and an LSB for lower extremity-related symptoms. The choice for these ganglions revolves around the anatomical relationship of visceral sympathetic-mediated activity stemming predominately from the T1 to L2 spinal levels. The T1-L2 ganglions are unique in that they are the only set of ganglions to contain white rami which serve as a relay station for paravertebral and prevertebral ganglion as well as receiving general visceral afferent activity [1]. Each spinal level correlates to different organ systems [8], and therefore, due to this relationship, nerve blocking of the ganglion, particularly in the thoracic region, could produce unpredictable autonomic responses [1]. For this reason, we believe that blocking at the L3 spinal level will insure no visceral involvement. 

## Case presentation

Review of the case

A young Caucasian male with no past medical history presented to the clinic with chronic neuropathic symptoms of the left lower extremity refractory to previous interventions. The patient reported symptoms of paresthesia localized to the left foot for over 18 months. The symptoms were diffuse and not localized to a specific dermatome. They were described as a constant "tingling". The patient had no sensory deficits to the symptomatic foot, and full strength was observed. Tinel's test at the tarsal tunnel was negative. Previous diagnostic studies including MRI and nerve conduction studies were unremarkable. The patient reported minimal improvement with venlafaxine and gabapentin. Dry needling, transcutaneous electrical nerve stimulation (TENS) unit therapy, and osteopathic manipulative medicine aimed at decreasing fascial tension also resulted in minimal benefit. The patient additionally complained of mild sacroiliac (SI) joint and piriformis tenderness at the time of presentation. He opted to undergo a diagnostic left LSB with the idea to observe whether the atypical neuropathic symptoms he was presenting with would be affected by the block. 

Prior to the completion of the LSB, to investigate a proposed mechanism of therapeutic benefit that we hypothesized to be playing a role in the clinical significance of LSB(s), a series of diagnostics were completed. A point-of-care ultrasound (POCUS) of the posterior tibial artery (PTA) was performed to measure blood flow and arterial dilation. Capillary refill of the hallux was measured with the mean average taken over three separate measurements. Lastly, the temperature of the plantar aspect of the foot was measured. It was then planned that immediately following LSB, the patient would again undergo POCUS at a marked location of the PTA just superior to the tarsal tunnel to ensure the same location of the artery was being measured. This ended up being approximately 30 minutes following the block. Capillary refill and temperature would be re-measured as well.

**Figure 1:**
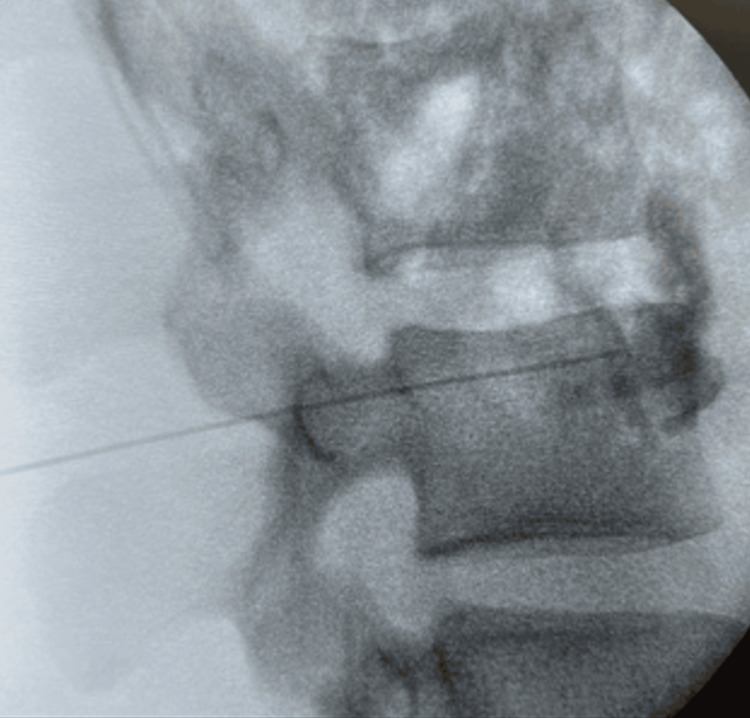
The vertebral body of L3 and the confirmation of the needle within the ganglion via contrast.

The patient underwent a left LSB consisting of 10 cc of .25% bupivacaine. This was completed at the level of L3 as current practice suggests the ganglion is largest at the L2/L3 spinal level. We further believe that through our understanding of autonomics, there is no visceral innervation from the sympathetic ganglion at the level of L3 making it a safer target. Confirmation of needle placement within the ganglion was observed via contrast (Figure 1).

**Figure 2:**
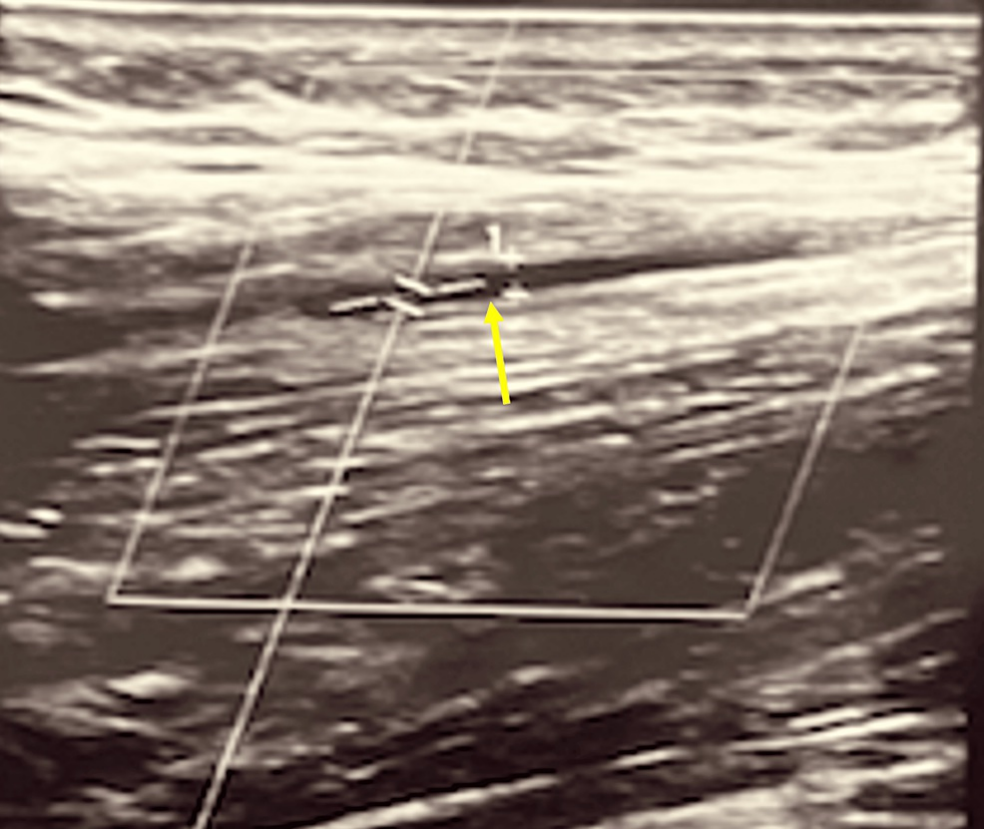
Pre-LSB: the PTA measures .17 cm in diameter with a blood flow velocity of 65.1 cm/s. LSB: lumbar sympathetic block; PTA: posterior tibial artery

**Figure 3:**
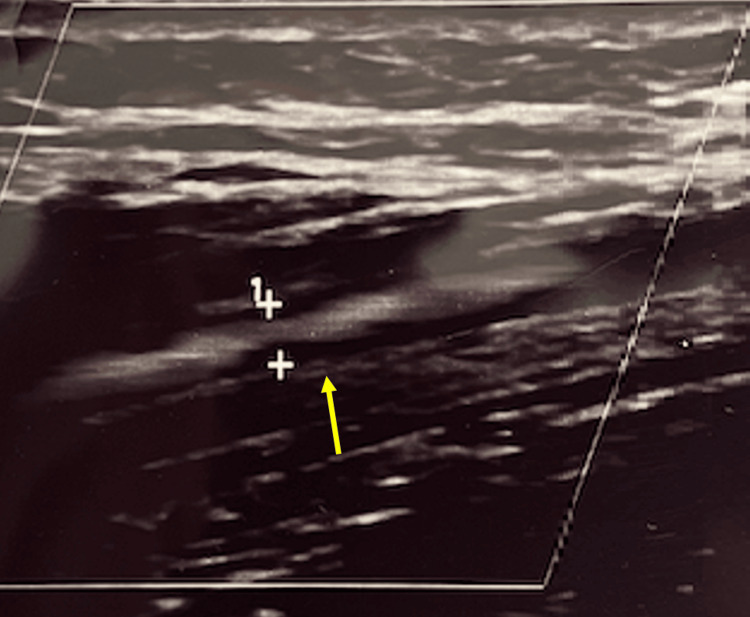
Post-LSB: the PTA measures .27 cm in diameter with a blood flow velocity of 57.4 cm/s (note Figure 1 and Figure 2 are not to scale and Figure 2 is with a Doppler) LSB: lumbar sympathetic block; PTA: posterior tibial artery

Following the block, the results were compared. Prior to the LSB, the PTA measured .17 cm in diameter (Figure 2) before then dilating to .27 cm following the LSB (Figure 3). Arterial blood flow velocity measured 65.1 cm/s pre-LSB before decreasing to 57.4 cm/s 30 minutes post-LSB.

Capillary refill was measured at x̅ 3.92 seconds pre-block and x̅ 1.30 seconds immediately post-block. The temperature of the foot was measured at 34.4 degrees Celsius pre-block and increased subsequently to its maximal temperature increase at 37.2 degrees Celsius seen at 90 minutes post-block.

**Figure 4:**
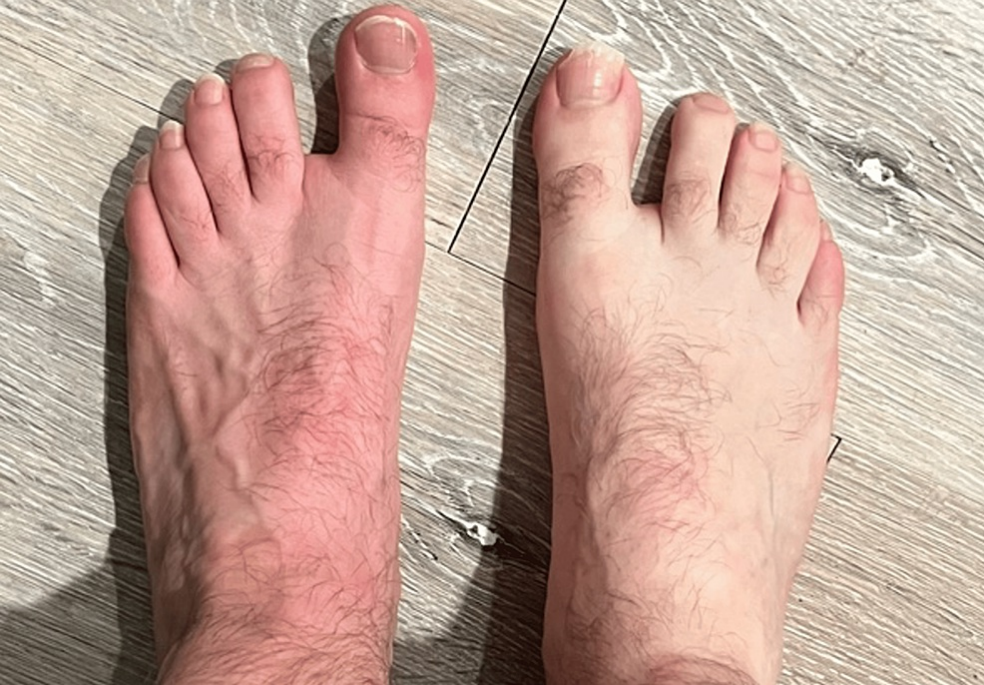
Significant erythema and dilation of dorsal veins on the ipsilateral foot of the LSB. LSB: lumbar sympathetic block

The patient subjectively experienced decreased pain, paresthesia, and an increase in sensation in the symptomatic foot approximately 45 minutes post-LSB. The left foot was observed to have a notable increase in erythema compared to the right foot (Figure 4). While not specifically measured, the patient did also report a decrease in perspiration of the left foot in comparison to the right. Lastly, the patient's left SI joint and piriformis felt subjectively better in the following days post-LSB.

The subsequent morning following the block, the patient noted the observed erythema had decreased suggesting the anesthetic used within the block had worn off. 

## Discussion

It's proposed that lumbar sympathectomy produces a vasodilatory effect by decreasing sympathetic tone [7]. Through POCUS, it was shown that, following LSB, significant arterial dilatory effects were seen in the PTA (Figure 2 and Figure 3). This increased blood flow, in theory, would allow for improved tissue perfusion and healing following LSB. This concept expands the utility of an LSB beyond just pain reduction. Matarazzo et al. showed that more than 385 patients who had undergone surgical sympathectomy for peripheral vascular disease had not just improvements in pain but healing of trophic lesions [9]. This finding further suggests the sympathetic role in vasoconstriction. These findings, if sustained over time, could show how LSB could serve as a potential therapeutic treatment modality for patients experiencing various ulcers and wounds refractory to treatment.

Though sympathetic pain is most notably associated with CRPS, sympathetic activity has been found to be associated with a variety of conditions such as fibromyalgia, temporomandibular disorder, arthritis, and back pain, to name a few [10,11]. Here, we presented a case of a patient who also complained of chronic SI joint pain and piriformis tendinosis that subjectively improved following LSB. While the pain relief appeared to be short-lived and not the complete scope of reasoning for this injection, this further helps support the role of sympathetic-mediated pain in chronic conditions.

LSBs have previously been reported to be deemed successful with a 2-3-degree Celsius temperature rise in the associated extremity [12]; however, it should be noted that false negatives may be observed in those with peripheral artery disease [13]. While these studies used much more controlled environments and advanced technology for temperature measurement, the patient presented in our case had a rise in temperature of 2.8 degrees Celsius in the ipsilateral foot. This not only helps confirm that the block was successful but furthers the significance of the PTA dilation and increased blood flow observed via POCUS post-LSB. LSBs' success can also be related to decreased perspiration, hence their role in treating hyperhidrosis [1]. While not objectively measured, our patient also reported decreased perspiration compared to the contralateral side following LSB.

Pathophysiology of sympathetic pain

**Figure 5:**
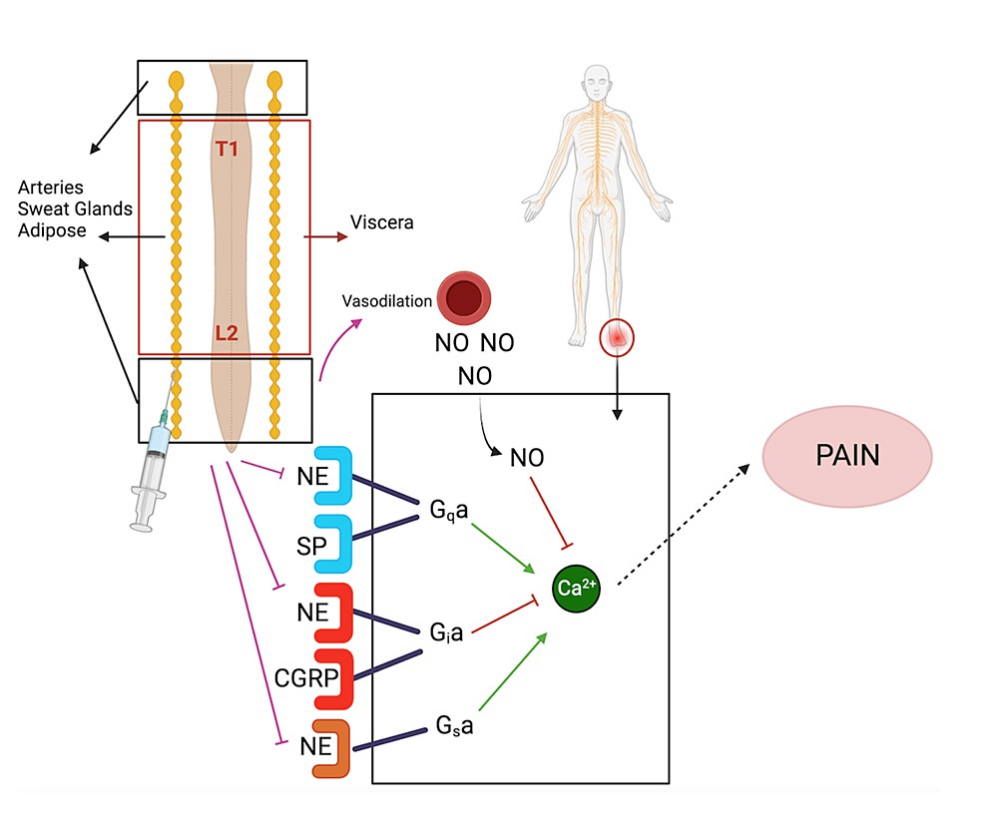
Pathophysiology of sympathetic pain This image was created by Zachary Dickey via BioRender. NE: norepinephrine; SP: substance P; CGRP: calcitonin gene-related peptide; NO: nitric oxide; G_q,I,s_α: G-coupled protein cell receptors

There are a prolific number of pathways and molecular mechanisms associated with pain; however, here, we look to explore the direct relationships of pain and the SNS (Figure 5). While the exact mechanism remains unclear, it’s thought that various neuropeptides such as calcitonin gene-related peptide (CGRP), substance P, and norepinephrine can maintain sympathetic-induced pain [14]. CGRP is a derivative of the dorsal root ganglia (DRG), is primarily located in afferent neurons, and is directly related to the excitatory effects of substance P [14]. This could explain how Iwase et al. were able to show decreased DRG activity in a rat model following sympathectomy [10]. During heightened sympathetic activity, there is increased sensitivity of peripheral pain receptors to the principal adrenergic neurotransmitter norepinephrine, which not only interacts with pain receptors but also causes vasoconstriction, disrupting proper blood flow [14,15]. Interestingly, nitric oxide (NO) may reduce pain signaling via the cyclic guanosine monophosphate (cGMP) pathway [14,16]. Therefore, restoring proper blood flow alone may improve pain. Furthermore, stimulation of two receptors, which also play a role in arterial dilation, reduces sympathetic activity and increases vagal tone [17]. Furthermore, NO can activate Gi receptors, causing the inhibition of pain transmission [14]. Norepinephrine is peculiar in that it can stimulate two receptors, potentially explaining the role of serotonin-norepinephrine reuptake inhibitors (e.g., venlafaxine) in decreasing pain, but additionally activate Gs receptors, activating pain transmission [14].

While certainly the pathophysiologic picture is much larger than this, these pathways help explain how an LSB contributes to the potential amelioration of pain. While it's hard to objectively measure how LSB can reduce pain, our ability to show significant arterial dilation and the understanding of these mechanisms may help explain the relationship. Certainly, future studies should be aimed to further investigate this proposed relationship and mechanism.

Autonomics and the future

As we better understand the role of autonomics and the corresponding spinal cord levels to visceral organs (Table 1), we can target those various spinal levels for therapeutic benefits. While not directly blocking the sympathetic chain ganglion, neuromodulation is growing in clinical significance based on this theory. Similar to sympathetic blocking, neuromodulation can decrease SNS activity by stimulating the dorsal column of the spinal cord, leading to an inhibition of the sympathetic pathways as they reach the spinothalamic tract [18]. By utilizing the viscera's relationship to spinal levels, the placement of a spinal cord stimulator, for example, can decrease refractory angina pectoris [18]. Similarly, neuromodulation has been explored in patients with heart failure and animal models for ventricular arrhythmias [19]. While more clinical evidence is certainly needed in this field, utilizing the autonomic relationship to structure and function allows one to explore potential treatment modalities for gastrointestinal, genitourinary, or gynecological pathologies. This understanding and relationship open the door for innovative treatment modalities, but also allow us to understand why neuromodulation or sympathetic blocks in different areas of the spinal cord could produce various autonomic responses. For example, a bilateral sympathetic block could result in erectile dysfunction or impotence [1]. The utilization of a stellate ganglion block for the treatment of post-traumatic stress disorder (PTSD) [20] shows the broad spectrum of investigation of sympathetic blockade.

**Table 1:** Sympathetic visceral somatic relationships

Organ/region	Spinal level	Organ/region	Spinal level
Head and neck	T1-T4	Kidneys	T10-T11
Heart	T1-T5 (left)	Upper ureter	T10-T11
Lungs	T2-T7	Lower ureter	T11-L1
Foregut	T5-T9	Bladder	T11-L2
Midgut	T10-T11	Gonads	T10-T11
Hindgut	T12-L2	Uterus/cervix	T10-L2
Appendix	T10-T12	Prostate	T12-L2
Upper extremity	T2-T8	Lower extremity	T11-L2

## Conclusions

While sympathetic nerve blocks have long been utilized for various clinical conditions, sympathetic-meditated pain is multifaceted and not completely understood. Here, we were able to present a case of a patient who underwent a diagnostic LSB and received neuropathic and chronic musculoskeletal pain relief. The case was also able to show and quantify significant arterial dilation following the LSB where we then proposed various mechanisms of therapeutic benefit through this increase in blood flow. There are various trials exploring the utility of blocking sympathetic activity, whether via ganglion block or neuromodulation. Regardless, future research and investigation is needed. Furthermore, a better understanding of the pathophysiologic mechanisms of sympathetic-mediated pain allows for the practical application of these various treatment modalities.
